# The role of Treg cells in colorectal cancer and the immunotherapy targeting Treg cells

**DOI:** 10.3389/fimmu.2025.1574327

**Published:** 2025-04-16

**Authors:** Hanqing Yu, Ruiliang Yang, Meixiang Li, Dan Li, Yuanqing Xu

**Affiliations:** ^1^ Department of General Surgery, The Sixth People’s Hospital of Huizhou, Huizhou, China; ^2^ Department of Internal Medicine, The Sixth People’s Hospital of Huizhou, Huizhou, China

**Keywords:** colorectal cancer, regulatory T cells, chemokines, immune suppression, immunotherapy, immune checkpoint inhibitor

## Abstract

Colorectal cancer (CRC) is among the most prevalent and lethal cancers globally, accounting for approximately 10% of all cancer cases and deaths. Regulatory T (Treg) cells, which accumulate in CRC tissue, suppress anti-tumor immune responses and facilitate tumor progression. This review discusses Treg cell origins and functions, along with the mechanisms by which Tregs influence CRC development. In addition, we highlight therapeutic strategies targeting Tregs-such as immune checkpoint inhibitors and combinatorial approaches-to enhance effector T cell responses. A deeper understanding of Treg-mediated immunosuppression in CRC may inform the design of more effective immunotherapies and precision medicine strategies.

## 1 Introduction

Colorectal cancer (CRC) is the third most common cancer worldwide, contributing to 10% of cancer cases and deaths globally (1). It is a heterogeneous disease, with genetic and molecular variations affecting clinical outcomes and treatment responses (2, 3). Approximately 70% of CRC cases are sporadic, while 20%-30% are familial, with one-third linked to highly penetrant genetic mutations, referred to as hereditary CRC. The remaining familial cases involve low-expressivity susceptibility genes and polymorphisms influenced by environmental or genetic factors (4–6). Chronic inflammation, particularly in inflammatory bowel disease (IBD), also increases CRC risk (7, 8). Colorectal polyps or adenomas, arising from epithelial cells, are precursors to CRC, where genetic and epigenetic changes lead to malignant transformation, progressing through the adenoma-carcinoma sequence (2, 3).

Host immune status significantly influences CRC development. Immune cells play a critical role in tumor immunity by suppressing immune effector cells in the tumor microenvironment (TME) (9–12), facilitating immune evasion and tumor growth (13–17). Studies show increased Treg infiltration in CRC tissues compared to normal tissues, correlating with higher TNM stages and elevated mRNA expression of Foxp3, IL-10, and TGF-β1 in tumor-infiltrating CD4+ T cells (18). Peripheral blood Treg levels are also higher in CRC patients, with greater Treg infiltration in tumor-adjacent tissues and regional lymph nodes than in distant or non-regional nodes (19, 20). Depleting Tregs can enhance cancer immunotherapy efficacy (21), but risks autoimmune diseases by disrupting immune tolerance (22, 23). Effective immunotherapy should selectively target TME Tregs while preserving peripheral Tregs to avoid autoimmune responses (24–26).

While CRC treatment primarily relies on surgery, radiotherapy, and chemotherapy, targeted therapies and immunotherapies are increasingly utilized, advancing “immuno-precision medicine.” Tregs typically promote tumor progression by suppressing anti-tumor immunity but may also exert anti-inflammatory protective effects, particularly in early tumorigenesis, where excessive inflammation could drive malignancy (27). The dual roles of Tregs in CRC remain incompletely understood, necessitating further research to develop context-specific immunotherapeutic strategies. This review introduced the role of Treg cells in CRC and progress in immunotherapy targeting Treg cells.

## 2 Origins and classification of Treg cells

Treg cells are classified based on their developmental origins into natural regulatory T cells and peripherally induced regulatory T cells (28–31). Thymus-derived Treg cells, formally known as naturally occurring Treg (nTreg) cells, develop in the thymus from CD4^+^ single-positive thymocytes and are considered to exhibit a high affinity for self-peptide major histocompatibility complex (MHC) molecules through their T cell receptor (TCR) (32). In contrast, peripheral Treg (pTreg) cells are generated in the periphery following antigen encounter under the influence of various factors, such as IL-2 and TGF-β (33). The pathways for the generation of Treg cells in the TME differ from those in normal tissues. In the TME, multiple factors interact to influence immune cells generation (34, 35). Chemokines recruit Treg cells from the thymus, bone marrow, lymph nodes, and peripheral blood to the tumor site, where they proliferate. Dysfunctional antigen-presenting cells can also induce the differentiation and proliferation of Treg cells. Moreover, suppressive molecules in the TME can directly convert CD4^+^CD25^+^ T cells into CD4^+^CD25^+^ FoxP3^+^ Treg cells (36).

Treg cells are integral to immune tolerance and anti-inflammatory responses, comprising both CD4^+^ and CD8^+^ subtypes (37). Both subsets exhibit functional differences, with a prominent distinction in their recognition of antigens. CD8^+^ Treg cells recognize antigens presented by MHC-I molecules, which are expressed on nearly all nucleated cells, enabling them to be activated by virtually any cell and exert inhibitory functions. In contrast, CD4^+^ Treg cells are activated only by cells expressing MHC-II molecules (37). Foxp3, a member of the forkhead transcription factor family, is a key intracellular marker and the principal regulatory factor of Treg cells (38, 39). Human Foxp3^+^ CD4^+^ T cells can be classified into three subsets based on the expression of CD4, CD45RA, CD25, and Foxp3: naïve/resting Treg cells, effector/activated Treg (eTreg) cells, and non-Treg cells. Although Foxp3^+^ T cells can be induced from conventional T cells via TCR stimulation, these induced cells typically secrete inflammatory cytokines and lack immunosuppressive capabilities. In contrast, specific cytokines or microbiota can promote the differentiation of CD4^+^CD25^-^ T cells into functional Treg cells with immunosuppressive properties. Conversely, certain cytokines or specific microbiota can induce Treg cells with immune-suppressive functions from CD4^+^CD25^-^T cells. In contrast, non-Treg cells do not possess immune-suppressive functions but instead produce inflammatory cytokines, such as interferon (IFN)-γ and IL-17 (36, 40).

## 3 Function of Treg cells

Treg cells possess two major functional characteristics: immune tolerance and immune suppression. Immune tolerance refers to the inability of Treg cells to respond to high concentrations of interleukin (IL)-2 stimulation alone, solid-phase coating, or soluble anti-CD3 monoclonal antibodies (mAb), as well as the lack of response to combined CD3 and CD8 monoclonal antibody stimulation. Additionally, Treg cells themselves do not secrete IL-2. Immune suppression refers to the ability of activated Treg cells to nonspecifically suppress the activation and proliferation of T cells through various mechanisms, including inhibition of both CD4^+^ and CD8^+^ T cells.

The immune suppressive function of Treg cells is mediated through multiple mechanisms, including downregulation of co-stimulatory signals (41), IL-2 consumption, secretion of immunosuppressive cytokines (e.g., IL-10 and IL-35) (42, 43), and production of immunosuppressive metabolites. Cytotoxic T-lymphocyte-associated protein 4 (CTLA-4) is crucial for Treg cell-mediated suppression, as it prevents aberrant autoimmune responses and excessive immune activation, thereby protecting the host from autoimmune attacks (44, 45). Treg cells also inhibit anti-tumor immune responses, especially tumor antigen-specific T cell responses (46). IL-2 is essential for effector T cell activation and survival. The high-affinity IL-2 receptor, comprising CD25 (α-chain), CD122 (β-chain), and CD132 (γ-chain), is expressed on most Treg cells, which consume IL-2 and limit its availability in the TME. This depletion impairs effector T cell activation (47). IL-10 and IL-35 further suppress T cell function by downregulating MHC and co-stimulatory molecules on antigen-presenting cells (APCs) (42).

Treg cells also exhibit granzyme-dependent immune suppression, releasing granzymes and perforins that promote cytolysis by NK cells and cytotoxic T lymphocytes (CTLs) (48). In the TME (49), Treg cells amplify in an antigen-specific manner, displaying distinct T cell receptor (TCR) repertoires compared to conventional CD4^+^ T cells (50). Recent studies have shown that Treg cells within the TME are highly activated and phenotypically differentiated (51). Treg cells strongly suppress anti-tumor immune responses through the function of hypoxia-inducible factor 1α (HIF-1α). In terms of metabolism, anti-tumor immune cells such as CD8^+^ T cells primarily utilize glycolysis for activation. Tumor cells consume glucose, reducing blood glucose levels in the TME (Warburg effect), thereby inhibiting the activation process of CD8^+^ T cells (52). Meanwhile, Treg cells proliferate and exert their immunosuppressive functions by metabolizing abundant lactate (53) and fatty acids (54) in the TME. In glucose-enriched TME, such as in liver metastatic lesions, Treg cells are activated by large amounts of lactate. Consequently, Treg cells acquire high programmed cell death protein-1 (PD-1) expression, and their activation is further enhanced by anti-PD-1 monoclonal antibody (mAb) treatment, leading to resistance to PD-1/PD-L1 blockade therapy (55). Besides, lymphocyte activation gene-3 (LAG-3) and T-cell immunoglobulin and mucin-domain containing-3 (TIM-3) are emerging immune checkpoints that can further contribute to T cell exhaustion and Treg-mediated immunosuppression in CRC (56).

## 4 Role of Treg cells in CRC

In CRC, Tregs heighten their suppressive function by elevating surface molecules such as CD39, CTLA-4, and PD-1 (57, 58), and by secreting IL-10 and IL-35, which jointly modulate the B-lymphocyte-induced maturation protein 1 (BLIMP1) inhibitory axis in CD4^+^ and CD8^+^ TILs (59). Tregs also dampen T cell recruitment to tumor sites by reducing CXC chemokine ligand 10 (CXCL10) (60) and can regulate CRC cell growth via IL-6 modulation (61). Additionally, by suppressing Th1 cell activity, Tregs indirectly or directly promote CRC angiogenesis through the inhibition of Th1-derived angiogenesis inhibitors, such as transforming growth factor-beta (TGF-β), and the production of angiogenic factors like neuropilin-1 (NRP-1) (62, 63). Foxp3^+^ Treg-derived IL-10 promotes lung metastasis in CRC by acting on both Foxp3^+^ Tregs and myeloid cells (64). However, the role of IL-23R signaling in Treg cells has been found to differ significantly between murine models of sporadic and inflammation-associated CRC. Inflammatory factors are important in diseases’ development and progression (65–68). In inflammation-related CRC, IL-23R signaling in Tregs suppresses carcinogenesis, whereas in sporadic CRC, it facilitates tumor development. These findings underscore the importance of the underlying etiological factors in CRC, which may have distinct impacts on the disease’s progression (69).

Despite the fact that the immunosuppressive role facilitates tumor cell evasion of anti-tumor immunity (70–73), some studies suggest that, at least in the early stages of inflammation-associated tumorigenesis, Treg cells may inhibit tumor progression by suppressing inflammatory responses (74). However, the impact of FoxP3^+^ Tregs on CRC prognosis remains controversial. Some studies indicate a correlation between Treg infiltration and favorable prognosis (75), while others suggest an association with poor prognosis (76–78). Saito et al. (76) reported that this discrepancy might be due to the existence of different FoxP3^+^ T cell subsets, namely eTregs and non-Tregs, which are difficult to distinguish through immunohistochemistry. Compared to CRC dominated by non-Treg infiltration, CRC patients with high eTreg infiltration exhibit worse prognosis.

**Figure 1:**
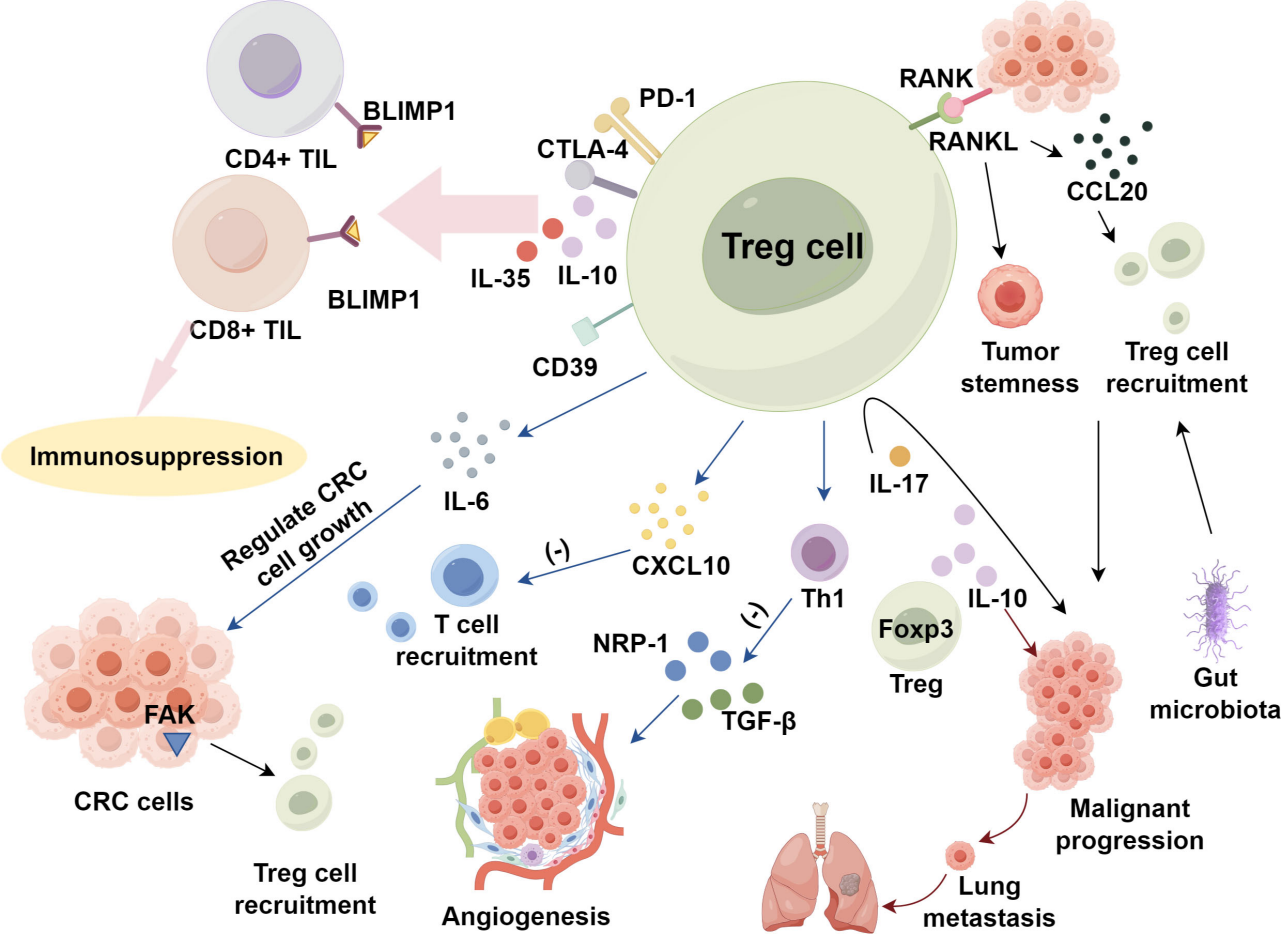
Regulatory T cells activation and expansion within tumor metabolic environment in CRC.

CRC cells and the TME further regulate Treg accumulation by secreting chemokines and cytokines (79). Focal adhesion kinase (FAK) in CRC cells enhances Treg recruitment (80). While the signaling of TNF receptor superfamily member 11a (TNFRSF11a, RANK) and its ligand TNF receptor superfamily member 11 (TNFRSF11, RANKL) induces CRC cells to produce CC motif chemokine ligand 20 (CCL20), leading to Treg recruitment through CCL20-CCR6 pathway and promoting tumor stemness and malignant progression (81). IL-17 directly activates Tregs, enhancing their maturation and functionality. This signaling mechanism forms a negative feedback loop that regulates inflammation, which contributes to cancer progression in CRC (82). Furthermore, gut microbiota may participate in immune suppression by promoting Treg accumulation (83) (**Figure 1**).

## 5 Targeting Treg cells in CRC immunotherapy

Peripheral immune tolerance mediated by Treg cells presents a significant challenge in immunotherapy. Therefore, to eliminate the immunosuppressive activity of Tregs during treatment, strategies such as depleting or inhibiting Tregs are often considered. These approaches aim to enhance the activity and accumulation of effector T cells, thereby achieving the therapeutic goal of immunotherapy. Studies have indicated that since the 20th century, researchers have attempted to treat cancer through Treg depletion, using low-dose cyclophosphamide or targeting specific surface markers such as CD25 (36). Immune checkpoint inhibitors have emerged as a prominent area of research in recent years, demonstrating substantial efficacy in various cancers, including metastatic MSI-H/dMMR CRC (84, 85). It has been confirmed that Tregs can express several surface markers, including CTLA-4, PD-1, and its ligand PD-L1, further highlighting the importance of targeting Tregs in immunotherapy (86). Moreover, combinatorial therapies targeting Treg surface receptors, such as glycoprotein A repetitions predominant (GARP), are under investigation (87, 88).

### 5.1 Immune checkpoint inhibitors

ICIs, such as CTLA-4 and PD-1/PD-L1 inhibitors, are widely used in cancer treatment, with MSI-H/dMMR identified as a biomarker for their efficacy. CTLA-4, expressed on activated T cells, inhibits T cell activation by competing with CD28 for CD80/CD86 binding, thereby promoting tumor cell survival. Anti-CTLA-4 therapy, like ipilimumab and temelimumab, induces Treg depletion through antibody-dependent cytotoxicity, enhancing T cell proliferation and antitumor immunity (58). These inhibitors are primarily used for metastatic melanoma, non-small cell lung cancer, renal, and bladder cancers, and in CRC, they are often combined with PD-1/PD-L1 inhibitors (86, 89).

PD-1, interacting with its ligands PD-L1 and PD-L2, suppresses T cell function, contributing to T cell exhaustion (36, 90, 91). PD-1/PD-L1 inhibitors, including pembrolizumab, nivolumab, durvalumab, atezolizumab, and avelumab, can restore effector T cell function and increase the CD8^+^ Treg ratio to negatively regulate Treg numbers (86). Recent clinical studies have shown that metastatic MSI-H/dMMR CRC is more likely to benefit from PD-1 inhibitors, whereas microsatellite stable (MSS)/mismatch repair proficient (pMMR) CRC shows minimal response (84). Andre et al. (92) evaluated pembrolizumab as a first-line treatment compared with standard chemotherapy in metastatic dMMR CRC, showing that pembrolizumab improved median progression-free survival (PFS), with fewer grade 3-5 drug-related adverse events than standard chemotherapy. In CRC, targeting LAG-3 or TIM-3 in combination with PD-1 or CTLA-4 blockade could potentially synergize to overcome Treg-mediated immunosuppression, thereby enhancing antitumor immunity (86, 93). These newer checkpoint molecules represent promising targets for combination immunotherapies aiming to modulate Tregs in the TME.

Several clinical studies have investigated low-dose ipilimumab combined with nivolumab in metastatic MSI-H/dMMR CRC, reporting objective response rates of 55-69%, with improved PFS and overall survival (OS) compared to PD-1 inhibitors alone (87, 94). The combination of low-dose ipilimumab and nivolumab has demonstrated robust and durable clinical benefits, potentially providing a new first-line treatment option for metastatic MSI-H/dMMR CRC patients. Additionally, combining PD-1 inhibitors with vascular endothelial growth factor receptor (VEGFR) inhibitors offers another therapeutic option for CRC. Activation of VEGF and its receptor VEGFR is associated with the immunosuppressive tumor microenvironment, contributing to tumor immune evasion by upregulating inhibitory immune checkpoint expression and recruiting immunosuppressive cells, including Tregs and myeloid-derived suppressor cells (95). A retrospective study on the use of VEGFR inhibitor regorafenib combined with PD-1 inhibitors in MSS/pMMR CRC patients revealed that while no objective responses were observed, the disease control rate was 78.3% (96). PLCG2 is an important oncogene and prognostic biomarker (97). Targeting PLCG2 can inhibit tumor progression, regulate the tumor immune microenvironment, and enhance immune checkpoint blockade therapy in CRC (98). Elevated TNF-α levels in the CRC tumor microenvironment promote the upregulation of CCR8 on Tregs via the TNFR2/NF-κB signaling pathway and FOXP3 transcription factor activity. Inhibition or depletion of TNFR2 significantly reduces the infiltration of CCR8+ Tregs, which in turn suppresses tumor progression and improves the efficacy of anti-PD1 therapy (99). In recent clinical trials (NCT04126733), regorafenib plus nivolumab have shown encouraging activity in CRC patients (100).

### 5.2 Photodynamic immunotherapy combined with IDO1 inhibition

To achieve efficient immunotherapy, Hu et al. designed a supramolecular prodrug nanocarrier for multiple immune modulators. By integrating photosensitizers, such as hyaluronic acid, magnesium phthalocyanine A, and indoleamine 2,3-dioxygenase 1 (IDO1) inhibitor NLC919 into the nanocarrier, the combination of photodynamic immunotherapy and IDO1 blockade led to a reduction in Treg numbers and inhibited tumor growth in a CRC mouse model (CT26), extending survival (101, 102). Further studies are needed to explore whether this nanoplatform can improve the efficacy of other immune modulators.

### 5.3 Targeting Treg surface receptors and markers

Glycoprotein A repetitions predominant (GARP) is expressed on the surface of activated Tregs, where it binds and activates TGF-β, playing a role in disease progression and immune evasion. Salem et al. (88) observed that Tregs lacking GARP had a reduced ability to suppress inflammation, leading to improved antitumor immunity and slower tumor progression in a colitis-associated colorectal cancer model. However, a study by Vermeersch et al. (103) in a murine colorectal cancer model showed that knocking out GARP did not delay tumor growth, suggesting that the absence of GARP is insufficient to affect Treg-mediated immunosuppressive activity. The potential of GARP inhibitors as a therapeutic target in CRC requires further investigation. Endoglin, an auxiliary receptor for TGF-β, is highly expressed on endothelial cells and cancer-associated fibroblasts. Schoonderwoerd et al. (104) reported that endoglin is highly expressed on Tregs in both murine and human CRC tissues, but absent from conventional CD4^+^ T cells. Anti-endoglin antibody (TRC105) has shown inhibition of angiogenesis and tumor metastasis in animal models of breast cancer (105). In CRC animal models, TRC105 treatment reduced Treg numbers in tumors, and the combination of TRC105 with PD-1 inhibitors significantly enhanced the efficacy of PD-1 inhibitors in CRC models (subcutaneous, orthotopic, and chemically induced) (104). The chemokine receptor CCR8 is selectively expressed on tumor-infiltrating Tregs. CCR8^+^ Tregs play a crucial role in the immune-suppressive tumor microenvironment in CRC by inhibiting the function of CD4^+^ Th and CD8^+^ T cells. Anti-CCR8 antibody therapy, targeting tumor-infiltrating CCR8^+^ Tregs, has the potential to restore the function of CD4^+^ Th and CD8^+^ T cells in CRC, thereby inducing antitumor immunity (106). Aspirin as an antitumor agent, influence key processes such as apoptosis, proliferation, metastasis, and senescence in cancer cells (107). Aspirin may help prevent colorectal cancer by modulating the levels of Enterococcus cecorum and TIGIT^+^ Treg cells, highlighting its potential therapeutic value in CRC treatment (108) (**Table 1**).

**Table 1:** Summary of Treg-targeting immunotherapy approaches in CRC.

Therapy	Agents	Mechanism	Clinical Relevance
Treg Depletion	Low-dose cyclophosphamide; Anti-CD25 antibodies	Reduces the number of immunosuppressive Tregs; Enhances effector T cell activity	Historically used to improve anti-tumor responses; May risk autoimmune reactions if peripheral Tregs are also depleted
Immune Checkpoint Inhibitors (ICIs)	ipilimumab, temelimumab; Anti- nivolumab; pembrolizumab; atezolizumab; durvalumab; avelumab.	Blockade of inhibitory receptors (CTLA-4, PD-1/PD-L1, LAG-3, TIM-3); Restores effector T cell function and proliferation; Depletes or functionally impairs Tregs	Demonstrates robust activity in metastatic MSI-H/dMMR CRC; Improves ORR, PFS, OS in MSI-H/dMMR CRC; Combination blockade shows promise in overcoming Treg-mediated immunosuppression
Photodynamic Immunotherapy+IDO1 Inhibition	Nanoplatform carrying photosensitizers plus IDO1 inhibitor (NLC919)	Photodynamic therapy triggers immunogenic cell death; IDO1 blockade curtails tryptophan depletion, limiting Treg proliferation	Reduction in Treg numbers and tumor burden in preclinical CRC models; Potential for combination with other modulators
Targeting Treg Receptors	Anti-GARP therapies; Anti-endoglin (TRC105); Anti-CCR8 antibodies; Anti-TNFR2 therapies	Selective inhibition or depletion of Tregs via blockade of unique molecules; Modulation of TGF-β activation and immunosuppressive signals	Anti-GARP can reduce Treg-mediated suppression; Endoglin blockade decreases Treg infiltration and augments PD-1 inhibitor efficacy; CCR8^+^ Tregs are major immunosuppressive cells in CRC; anti-CCR8 therapy restores Th and CD8^+^ T cell function
Aspirin and Microbiota Modulation	Aspirin; Manipulation of gut microbiota (e.g., *Enterococcus cecorum*)	Aspirin may influence Treg-associated pathways and gut microbiota composition; Reduces pro-tumoral inflammation and Treg-mediated suppression	Potentially lowers Treg levels or alters Treg phenotype; Demonstrated benefit in CRC prevention and therapy in some studies

## 6 Conclusion

Treg cells play a pivotal role in the immune landscape of CRC, facilitating tumor progression by suppressing anti-tumor immune responses and promoting tumor immune evasion. Their accumulation within the TME is driven by various factors, including chemokine signaling, cytokine modulation, and interactions with CRC cells. While Treg cells may offer protective effects in the early stages of inflammation-associated tumorigenesis, their overall contribution to CRC progression is largely detrimental. The dual nature of Treg cell functions, including both immune suppression and regulation of inflammatory responses, underscores the complexity of targeting Tregs in CRC therapy. Future research is urgently required to disentangle how Tregs switch from an anti-inflammatory role to a predominantly pro-tumor function over the course of CRC development. Elucidating the molecular and cellular underpinnings of this transition could pave the way for innovative immunotherapies that selectively counteract the immunosuppressive properties of Tregs while preserving their beneficial impacts on early-stage inflammation. Such targeted strategies hold the promise of improving clinical outcomes and advancing precision medicine in CRC. Besides, the development of therapies aimed at selectively depleting or inhibiting Treg cells within the TME, while preserving their peripheral functions, holds great promise for enhancing the efficacy of immunotherapy in CRC. Future research should focus on identifying specific Treg cell subsets, optimizing therapeutic strategies, and understanding the interplay between Treg cells and other immune components in the TME to achieve better clinical outcomes for CRC patients.
